# Separation of Drugs by Commercial Nanofiltration Membranes and Their Modelling

**DOI:** 10.3390/membranes12050528

**Published:** 2022-05-17

**Authors:** Vignesh Nayak, Jiří Cuhorka, Petr Mikulášek

**Affiliations:** Institute of Environmental and Chemical Engineering, Faculty of Chemical Technology, University of Pardubice, Studentská 573, 532 10 Pardubice, Czech Republic; cuhorka@seznam.cz (J.C.); petr.mikulasek@upce.cz (P.M.)

**Keywords:** nanofiltration, Spiegler–Kedem model, caffeine, paracetamol, naproxen

## Abstract

Pharmaceutical drugs have recently emerged as one the foremost water pollutants in the environment, triggering a severe threat to living species. With their complex chemical nature and the intricacy involved in the removal process in mind, the present work investigates the performance of commercially available polyamide thin-film composite tubular nanofiltration (NF) membranes (AFC 40 and AFC 80) in removing polluting pharmaceutical drugs, namely caffeine, paracetamol and naproxen. The structural parameters of the NF membranes were estimated by water permeability measurements and retention measurements with aqueous solutions of organic, uncharged (glycerol) solutes. The effect of various operating conditions on the retention of solutes by the AFC 40 and AFC 80 membranes, such as applied transmembrane pressure, tangential feed flow velocity, feed solution concentration and ionic strength, were evaluated. It was found that the rejection of drugs was directly proportional to transmembrane pressure and feed flow rate. Due to the size difference between caffeine (MW = 194.9 g/mol), naproxen (MW = 230.2 g/mol) and paracetamol (MW = 151.16 g/mol), the AFC 40 membrane proved to be efficient for caffeine and naproxen, with rejection efficiencies of 88% and 99%, respectively. In contrast, the AFC 80 membrane proved to be better for paracetamol, with a rejection efficiency of 96% (and rejection efficiency of 100% for caffeine and naproxen). It was also observed that the rejection efficiency of the AFC 80 membrane did not change with changes in external operating conditions compared to the AFC 40 membrane. The membrane performance was predicted using the Spiegler–Kedem model based on irreversible thermodynamics, which was successfully used to explain the transport mechanism of solutes through the AFC 40 and AFC 80 membranes in the NF process.

## 1. Introduction

Pharmaceutical drugs are a boon to mankind, have a huge positive impact on human well-being and prolong human life expectancy. These drugs act as lifesavers during times of crisis. However, overuse or untreated discharge of the same from pharmaceutical industries and other sources can have a detrimental effect on the environment and all life that depends on it. Pharmaceutical drugs can enter water sources via different pathways, such as municipal wastewaters, household waste, livestock waste, unpermitted dumping and non-regulated human sewage discharge [1,2]. These drugs are called pharmaceutically active compounds (PhACs) and are classified as emerging pollutants.

Emerging pollutants are those micropollutants that are difficult to degrade due to their complex structure and persist in the environment, causing harm to aquatic and other life forms [3,4]. Emerging pollutants are further classified into five major types, namely pharmaceuticals, personal care products, hormones, pesticides and industrial chemicals [5]. The discharge of these PhACs, including analgesics, antibiotics and anti-inflammatory drugs, has increased in recent times, and it is estimated that around 160 such PhACs are dumped into water bodies. Some of these are treated at municipal treatment plants, but the majority are allowed to pass unchecked [6]. Even though the concentrations of most of the PhACs are in µg/L or ng/L, the amplified intake of pharma products due to the increased human population has caused alarm and is becoming one of the principal water pollutants [7,8,9].

There has been no significant change in the regulatory guidelines for the discharge of these drugs, and most of them are discharged without adequate treatment [10,11]. Once eluted into the water system, PhACs are very difficult to remove, and they circulate in the water cycles and the food chain, eventually reaching the human body [12]. Long-term exposure to these drugs can have severe implications on human health related to kidney and heart disease and stroke, reproductive, gastrointestinal and cardiovascular issues and suppression in the endocrine system [13,14]. Cases of bacterial resistance in the environment, and genetic and systematic damage to some mussels and fish species, have also been identified [15].

Presently, even with all the advancements in pharmaceutical drug technology, such as complex chemical structures, low volatility and concentration, varying hydrophobicity and interactions with other solutes, it is nearly impossible for conventional wastewater treatment plants to efficiently eliminate them [1,16,17]. With the complexity of complete removal of PhACs from wastewater in mind, several techniques have been proposed, for example, biosorption [18], coagulation [19], photocatalysis [20], ozonation [21] and membrane separation [22], with the disadvantages of each limiting their use [6].

Nanofiltration has emerged as a major contender owing to its durability, effectiveness, ease of use and lower running cost [23,24]. Several reports have demonstrated the effectiveness of nanofiltration membranes. Quintanilla et al. [25] studied nine different types of PhAC using NF 90 and NF 200 membranes, giving insight into different mechanisms of rejection, such as size exclusion and electrostatic repulsion. Similarly, Arsuaga et al. [26] used NF 270 and NF 90 for the separation of sulfamethoxazole, diclofenac sodium, hydrochlorothiazide, 4-acetamidoantipyrine, nicotine and ranitinide hydrochloride, where NF 90 was superior in performance with over 95% rejection, and NF 270 achieved over 75% rejection for all the drugs. Here, the rejection was governed by steric hindrance and electric interaction at the solute/membrane interface. On the other hand, Verliefde et al. [27] subjected 21 different types of drug (neutral, negative and positive) to separation using Trisep TS80 TSF and Desal HL membranes to study the fouling effects.

Keeping this literature study in mind, we also considered our previous success with NF membranes as a foundation for this study; the authors previously investigated the rejection efficiency of AFC 30, 40 and 80 membranes for the removal of ibuprofen and diclofenac, demonstrating that the AFC 40 membrane has almost 100% rejection efficiency, which is mainly governed by the sieving effect [28]. The present research is extended to other PhACs which are widely used in day-to-day life, namely, caffeine, paracetamol and naproxen, which belong to the classes of central nervous system stimulants, analgesics and anti-inflammatories, respectively. The research focus is on extending the scope of the AFC NF class membranes and setting the selection parameters for drugs based on their characteristics. The experiments were carried out for all drug samples at different feed concentrations in the range of 5 to 20 mg/dm^3^ between transmembrane pressures ranging from 5 to 30 bar for the AFC 40 and 10 to 30 bar for the AFC 80 (due to its very low permeability at 5 bar). In addition, the pore size of the membranes was calculated using the steric-hindrance pore model proposed by Nakao and Kimura [29], which takes into consideration the steric hindrance effect on the transport of uncharged molecules [30]. The experimental rejection data were analysed using the Spiegler–Kedem model. The study aims to expand the application of tubular AFC 40 and 80 membranes for removal of various PhACs and evaluate the performance of the membranes for drugs with different characteristics such as molecular weight and charge.

## 2. Materials and Methods

### 2.1. Materials

The AFC tubular NF membranes were purchased from PCI membrane systems, Poland. The characteristics of the membranes are given in Table 1. The caffeine (MW = 194.19 g/mol) and naproxen sodium (MW = 252.24 g/mol) were obtained from Sigma Aldrich. Paracetamol (MW = 151.16 g/mol) was bought from Penta chemicals, Prague. These were used for standard solution preparation for the calibration curve. The feed solutions used during filtration experiments were prepared from tablets as follows: caffeine 200 mg tablet, parapyrex 500 mg from Dr. Max Pharma Ltd. and Nalgesin S 275 mg (naproxen natrium salt) from KRKA pharmaceutical company. The tablets were ground using mortar and pestle, followed by ultrasonication of the solution for 30 min. This solution was then filtered using a 0.7 µm glass filter in a dead-end filtration unit at 0.5 bar vacuum and diluted as per requirement. Other chemicals, such as glycerol and sodium chloride (NaCl), were supplied by Lach-Ner. All the solutions were prepared using distilled water with conductivity less than 10 μS cm^−1^.

### 2.2. Experimental Setup

**Figure 1:**
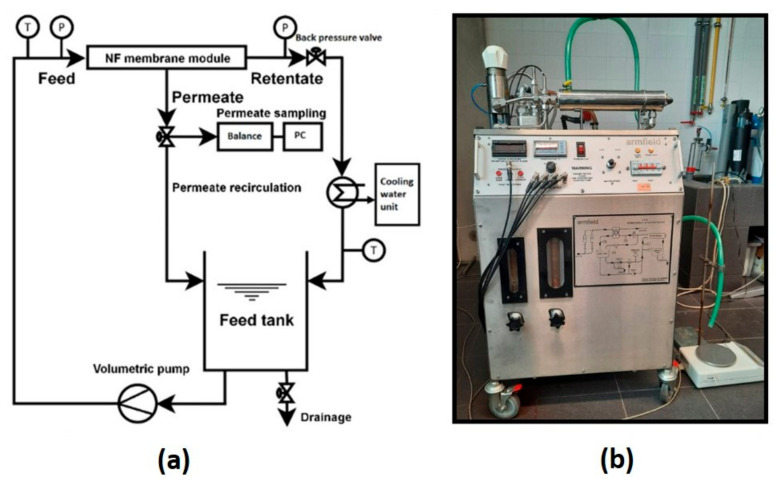
Experimental setup of the nanofiltration unit (**a**) diagram and (**b**) actual NF lab unit.

The NF unit consisted of a 10 dm^3^ feed tank equipped with an NF membrane module (FT 18, Armfield, GB, Ringwood, United Kingdom (UK)) and a cooling water unit (TAEevo, Armfield, GB, Ringwood, United Kingdom (UK)), which was used to carry out all the separation experiments (Figure 1). The temperature of the bulk feed solution was maintained at 25 ± 0.5 °C. The weight of the permeate was constantly measured with the aid of a weighing machine (Balance KERN KB, Balingen, Germany) which, in turn, was connected to a computer installed with software to calculate the flux. The concentration of the PhACs was measured using high-performance liquid chromatography (HPLC). In the case of NaCl rejection, the conductivity was measured using a tabletop inoLab Cond. 7110 m from WTW (Weilheim, Germany) equipped with a conductivity electrode TetraCon^®^ 325. The concentration of glycerol was calculated by total organic content analysis using the model Skalar Formacs HT/TN TOC/TN Analyzer (Breda, The Netherlands).

### 2.3. Analytical Methods for Caffeine, Paracetamol and Naproxen

The concentration of the PhACs was measured by HPLC from Agilent Technologies (model: 1260 infinity II Prime LC, Santa Clara, CA, United States (USA)) equipped with column Nucleosil 120 C18 (5 μm, 250 mm × 4 mm) and a diode array detector (DAD). The HPLC parameters for all three drugs are given below (Table 2), and all the analyses were conducted in isocratic mode.

### 2.4. Separation Methods

All the experiments for each drug were carried out on new membranes (i.e., 3 membranes for 3 drugs were used) at the beginning of the experiment, and the membranes were cleaned with distilled water after every separation experiment to remove any adsorbed solutes from the membrane surface. Every membrane underwent compaction before the start of the experiments at 1 bar above the highest operating pressure of the rejection experiments (in this case, 31 bar) for two hours. This was done to minimise the effect on flux and rejection values once the polymer chains in the membrane became hampered (which is irreversible) at high pressures. The PhAC separation was carried out individually, and conditions were optimised. HPLC samples were collected after one hour at each pressure change to stabilise the pressure and obtain steady-state flow. The permeate was continuously recirculated into the feed tank to maintain a constant feed concentration. Feed and retentate samples were collected, and the average was considered (as feed concentration) to evaluate the rejection. All the experiments were duplicated to minimise experimental error. Errors were found to be less than 5%. All experiments were carried out at a maintained temperature of 25 °C ± 0.5.

#### 2.4.1. Water Flux and NaCl Glycerol Rejection Studies

The permeate flux was calculated using Equation (1) for both NF membranes at pressures from 5 to 30 bar to investigate the effects of different feed solutions on the membrane flux:

(1) $$J=\frac{V_{p}}{A\Delta t}$$

where V_P_ (dm^3^) is permeate volume, Δt (h) is the time interval and A (m^2^) is the effective membrane surface area. The hydraulic permeability coefficient (Lp) of the membrane is the pressure-dependent slope of distilled water flux.

NaCl rejection was carried out for a feed concentration of 1 g/dm^3^ at a 15 dm^3^/min feed flow rate (the highest possible on the NF setup), while the other conditions were maintained at a constant. It should be noted that all the experiments, except for distilled water flux, were conducted after 96 h of exposure of the membranes to the feed solutions to eliminate any adsorption effect that might have taken place:

(2) $$R\;\left(\%\right)=\left(1-\frac{\mathrm{Cp}}{C_{f}}\right)\text{×}100$$

where C_P_ is the NaCl concentration in permeate, and C_f_ is the NaCl feed concentration.

In a similar manner, glycerol was separated (feed concentration of 0.5 g/dm^3^) to calculate the pore size of the NF membranes using the steric-hindrance pore model.

#### 2.4.2. Experiments with Caffeine, Paracetamol and Naproxen

The PhAC separation was carried out similarly to the uncharged solutes separation, where the solution was separated at 5 bar for four hours and soaked in the feed solution for four days. The feed solution was separated at 5 bar on alternate days for circulation purposes. The control experiment was conducted by maintaining the feed concentration and flow rate at 20 mg/dm^3^ and 15 dm^3^/min, respectively, at 25 °C ± 0.5. Feed, retentate (which represents feed at the end of the experiment) and permeates were collected and analysed using HPLC. Following that, each parameter was changed and studied. For instance, the effect of feed concentration was studied by changing the concentration from 5, 10 and 20 mg/dm^3^ while maintaining another parameter at a constant. Similarly, the feed velocity was altered from 5, 10 and 15 dm^3^/min, while maintaining the rest of the parameters at a constant. For the effect of ionic strength, NaCl was added to the feed solution (0, 5 and 10 g/dm^3^) at 20 mg/dm^3^ feed concentration (of the evaluated drug) and 15 dm^3^/min feed flow velocity, respectively. The characteristics of the PhACs is given in Table 3.

### 2.5. Data Analysis and Modelling

The rejection efficiency of the membranes for PhAC removal was calculated using Equation (2). However, it was observed that the concentration of the feed solution near the membrane surface (C_m_) was not the same as the concentration of bulk feed solution. This is because of the concentration polarisation phenomenon that occurs near the membrane’s active surface, leading to crowding of the retained solutes, which pile up. Hence, considering this, the intrinsic rejection of the membranes was calculated by the film model where the C_m_ is considered and can be calculated as follows:

(3) $$R_{\mathrm{int}}=\left(1-\frac{C_{P}}{C_{m}}\right)=\frac{R_{0}\exp\left(\frac{J}{k}\right)}{1-R_{0}\left[1-\exp\left(\frac{J}{k}\right)\right]}\;$$

where C_p_ is the permeate concentration, C_m_ is the solute concentration at the active membrane surface and J is the permeate flux. The parameter k is the mass transfer coefficient and is obtained from the Sherwood relationship in general, as described below:

(4) Sh=βReaScbdhLc

Here, the experimental conditions decide the coefficient values β, a, b and c. For our experimental condition (tubular membrane, turbulent flow), k is calculated:

(5) k=0.023Re0.875Sc0.25Di,∞dh

where Reynolds (Re), Schmidt (Sc) and Sherwood (Sh) numbers are defined as

Sh=kdhDi,∞ , Re=uρdhη , Sc=ηρDi,∞

where D_i,∞_ denotes the infinite dilution diffusion coefficient of the solute i, and η and ρ represent the dynamic viscosity and density of the aqueous solution, respectively. The parameter u represents fluid velocity in the channel with d_h_ as its hydraulic diameter (inner diameter in the present case). The viscosity and density were considered equal to water due to low solute concentrations.

These values were used in predicting the model for these separation experiments. The Spiegler–Kedem model was considered due to its simplicity, with the membranes being thought of as a black box, and factors, such as the separation mechanism, being irrelevant. This model includes only two factors, namely reflection coefficient (σ) and solute permeability (ω), and the rejection is given as follows:

(6) $$R_{\mathrm{int}}=\frac{\sigma \left(1-F\right)}{1-\sigma F},\;\mathrm{where},\;F=\exp\left.\left(-\frac{1-\sigma }{\omega }\right.\;\cdot J\right),$$

This model is validated by the non-linear factor χ^2^, which is solved as follows:

(7) $$\chi^{2}=\sum \frac{{\left(R_{\mathrm{real}}-R_{\mathrm{model}}\right)}^{2}}{R_{\mathrm{model}}}$$

Reflection coefficient (σ) and solute permeability (ω) values were also calculated in the case of uncharged solutes (glycerol) for the calculation of the pore size of the respective membranes. The parameters σ and ω can be calculated as follows:

σ_SHP_ = 1 − S_F_{1 + (16/9)q^2^}, where (8)

S_F_ = (1 − q)^2^(1 + 2q − q^2^) and q = r_s_/r_p_, (solute radius vs. pore radius) (9)

ω_SHP_ = D.S_D_.(A_K_/Δx), here, S_D_ = (1 − q)^2^ (10)

## 3. Results and Discussion

### 3.1. Distilled Water Flux

**Figure 2:**
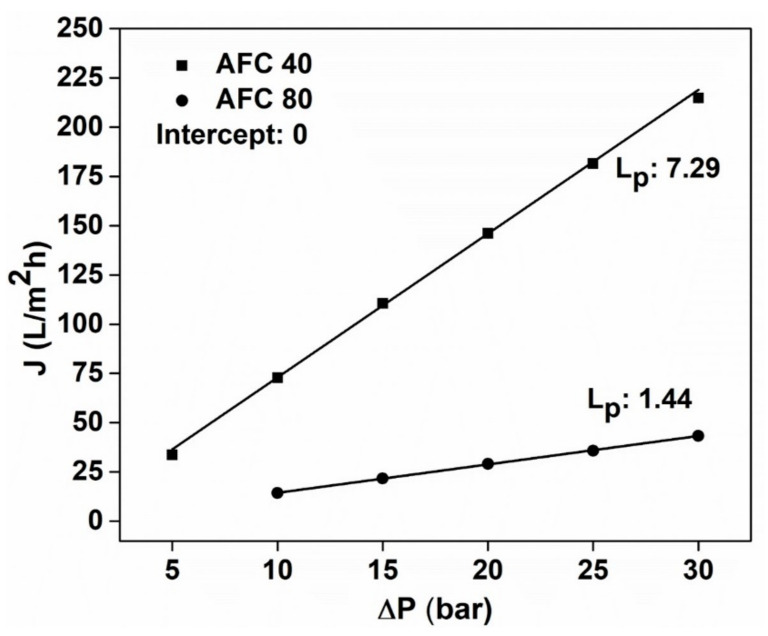
Distilled water flux of the AFC 40 and AFC 80 membranes at different operating transmembrane pressures.

The effect of applied pressure was studied on the distilled water flux, and, as expected, a linear increase in flux was obtained with every 5 bar increase in pressure (Figure 2). This result was consistent for both membranes, with a hydraulic permeability of 1.44 and 7.29 Lm^−2^h^−1^ for the AFC 80 and AFC 40 membranes, respectively. These values are similar to the results obtained in our previous study [29], and the deviation is less than 5%, which can be attributed to the different batches of membranes and experimental error. It can be observed from Figure 2 that the flux for the AFC 40 was more than four times the flux obtained for the AFC 80, which is due to its larger pore size, as calculated by the SHP model.

### 3.2. NaCl Rejection

**Figure 3:**
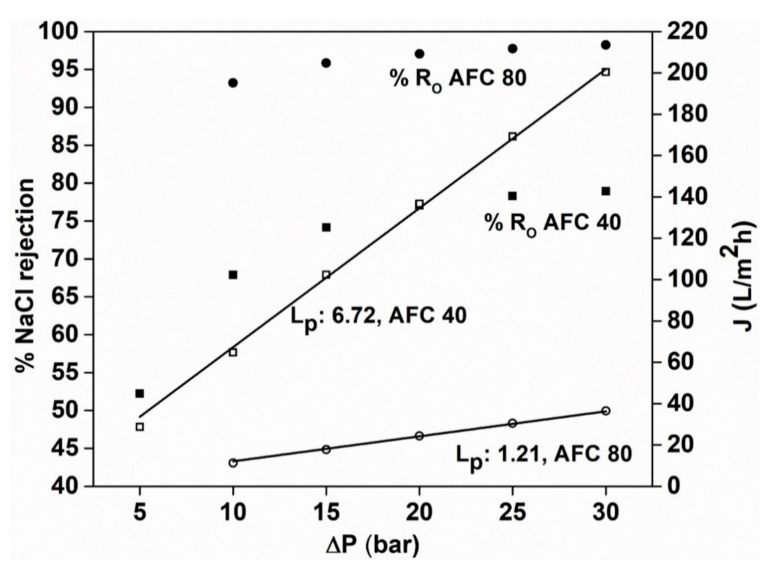
NaCl rejection for the AFC 40 and AFC 80 membranes.

Figure 3 presents the NaCl rejection and permeate flux values for both NF membranes. It can be seen that AFC 40 has a maximum rejection at 30 bar of ~79%, while AFC 80 has a maximum rejection of ~98%. This difference is due to the smaller pore size of the AFC 80, which can separate out NaCl solute molecules more effectively. In addition, it should be noted, that the negative charge of the membrane surface may contribute to the high rejection efficiency of the AFC 40 membrane by repelling the chloride ions, and the rejection is enhanced at higher pressure due to the higher flux rate.

It is important to mention that the flux was not significantly affected by the NaCl solute molecules in comparison to the distilled water flux (Figure 2); however, a slight decrease was seen. This is due to the lower concentration of NaCl feed solution (1 g/dm^3^), which did not cause any significant increase in the osmotic pressure on the feed side of the membrane and, thus, did not alter the driving force of the water molecules through the membrane. Therefore, based on the rejection values of NaCl, the AFC 40 membrane belongs to the loose NF range, whereas the AFC 80 membrane belongs to the tight NF range.

### 3.3. Glycerol Rejection and Pore Size Calculation

Despite having a higher molecular weight (92.09 g/mol) than NaCl (58.44 g/mol), glycerol was rejected by the AFC 40 and AFC 80 membranes by ~53% and ~94%, respectively. This is because, unlike in the case of NaCl rejection, glycerol is uncharged, and, hence, the separation takes place completely based on the sieving effect, without any interference from the negative charge present on the membrane surface. This was also proved by the superior permeate flux in the case of glycerol rejection in comparison to NaCl, which exactly matched the distilled water flux.

**Figure 4:**
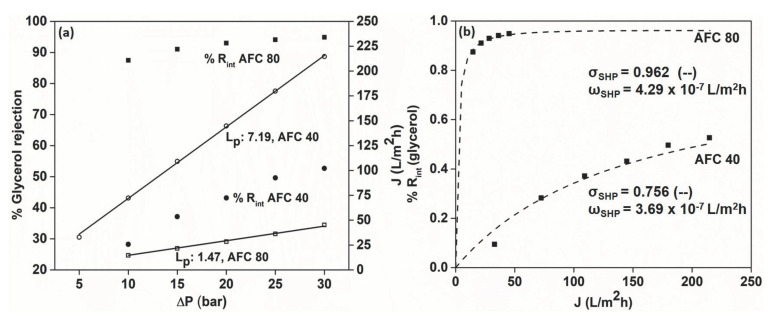
(**a**) Rejection of glycerol by the AFC 40 and AFC 80 membranes and (**b**) SHP model fit.

The observed rejection was used to calculate the intrinsic rejection values, which were then used in the SHP model fit presented in Figure 4b. A perfect alignment was observed for both the AFC 40 and AFC 80 membranes, with reflection coefficients (σ_SHP_) of 0.756 and 0.962, respectively. A higher reflection coefficient (closer to 1) means better retention capacity of the membrane [31]. These values were used to calculate the pore radius of the membranes and were found to be 0.283 nm and 0.345 nm for the AFC 40 and AFC 80 membranes, respectively.

### 3.4. Effect of Feed Concentration on the Rejection of PhACs

**Figure 5:**
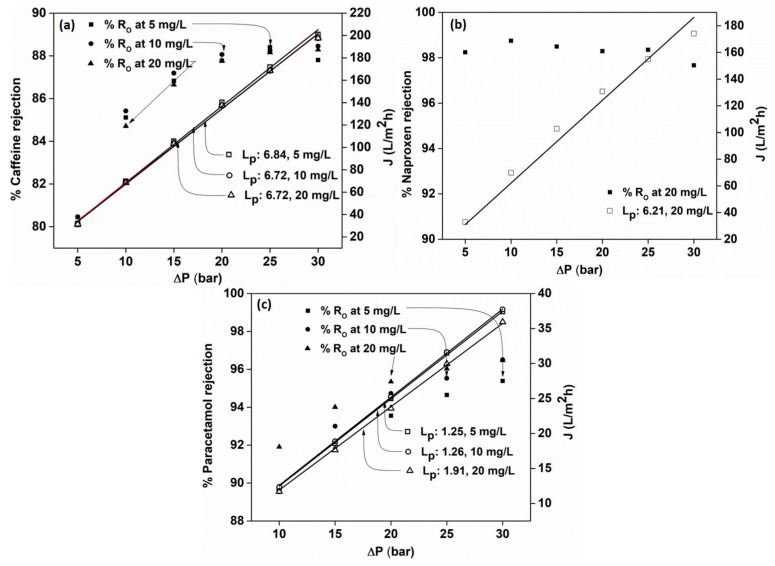
Effect of feed concentration on rejection efficiency of (**a**) caffeine, (**b**) naproxen and (**c**) paracetamol.

External operating parameters can have a huge effect on the rejection efficiencies of the membranes. In order to check the solute concentration effect, the membranes were put under test at different solute concentrations (5, 10 and 20 mg) for each drug residue. Caffeine (Figure 5a) and naproxen (Figure 5b) were rejected by the AFC 40 membrane, while paracetamol (Figure 5c) was rejected by the AFC 80 membrane. The increase in rejection from 5 bar to 30 bar pressure was due to the phenomenon called the ‘dilution effect’. This implies that the greater the permeate flux, the higher the rejection (as observed in the case of NaCl and glycerol separation).

Our investigation revealed that the retention of solutes increases linearly as the initial pressure rises from 5 to 20 bar. However, the optimum pressure for rejection lies between 25 and 30 bar, where the rejection hits a saturation point, and a plateau is observed, and no further increase is expected beyond this point. At higher pressure, the active surfaces of the membranes become saturated with solute ions near their surface pores and become non-responsive to any further increase in pressure. 

From Figure 5b, it is evident that naproxen is rejected more than caffeine by the AFC 40 membranes due to its higher molecular weight (230.2 g/mol) compared to caffeine (194.9 g/mol). Moreover, naproxen carries a partial negative charge which might be repelled by the negative charge on the membrane surface, as explained by several studies [32]. This is called the ‘Donnan exclusion principle’. However, based on the observed rejection pattern for caffeine, it could be concluded that the sieving effect plays a major role in the retention of drug residues. 

For paracetamol, the AFC 40 membrane showed a low rejection of 24% due to having the lowest molecular weight among the three (151.16 g/mol), and, hence, the AFC 80 membrane was chosen (rejection of 96% obtained). From Figure 5 it is evident that the effect of feed concentration on rejection efficiency, as well as the permeate flux, is negligible, with the observed maximum rejection of 85–88%, 98% and 95–96% for caffeine, naproxen and paracetamol, respectively. This is explained by the low solute concentration in the feed solution, which was incapable of causing any significant change in the osmotic pressure on the feed compartment and thereby incapable of influencing the driving force on the permeate side of the membrane (as observed for glycerol and NaCl rejection). In addition, the permeate flux was comparable to the flux of distilled water (Figure 2), which was within the effective limit of less than 5% experimental error. It should be noted that the AFC 80 membrane was not considered for caffeine and naproxen as it had a rejection efficiency of 100% (Note: As feed concentration does not significantly affect rejection efficiency, the effect of feed concentration on naproxen was not studied further).

### 3.5. Effect of Ionic Strength on the Rejection of PhACs

**Figure 6:**
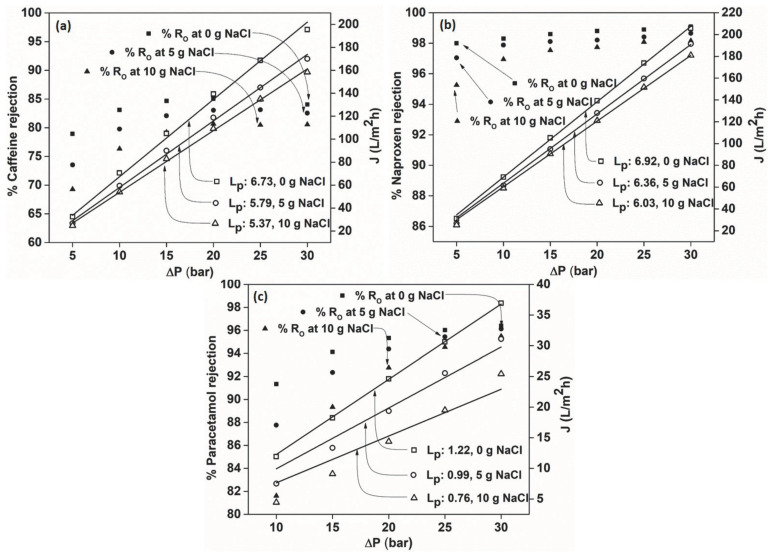
Effect of ionic strength on the rejection efficiency of (**a**) caffeine, (**b**) naproxen and (**c**) paracetamol.

Figure 6 presents the influence of ionic strength on the rejection efficiency, where NaCl acts as a precursor for the ionic effect. It can be seen that the permeate flux suffered with increasing NaCl concentration from 0 g/dm^3^ to 5 g/dm^3^ and 10 g/dm^3^ (Figure 6). However, the retention efficiency of the membranes for the PhACs was not altered and maintained a similar rejection pattern for rejection without NaCl. Nevertheless, when inspected closely, it was clear that the rejection was slightly lower, especially at low pressure from 5 to 20 bar, but, eventually, very similar rejection was obtained at higher pressure. In theory, the NaCl ions can form a shield near the membrane surface, which might be the cause of the slightly lower rejection values at lower pressures, where a higher percentage of drugs is transported by diffusion compared to the convection they experience at higher fluxes.

As discussed above, the rejection is mainly governed by the sieving effect and not charge, thus, having an insignificant impact on the retention capacity of the membranes. This was established by the similar rejection values obtained with and without NaCl solute ions. When the NaCl concentration was increased from 0 to 5 to 10 g/dm^3^, the osmotic pressure of the feed solution rose, explaining the change in permeate flux. This led to a decrease in the net driving force of the solvent, which governs the flux. However, this effect on rejection was negligible, as it did not alter the retention mechanism. This experimental study achieves relevance as drug residues are usually accompanied by other salts in wastewater that are present in the environment and can impact the treatment process.

### 3.6. Effect of Feed Flow Rate on the Rejection of PhACs

**Figure 7:**
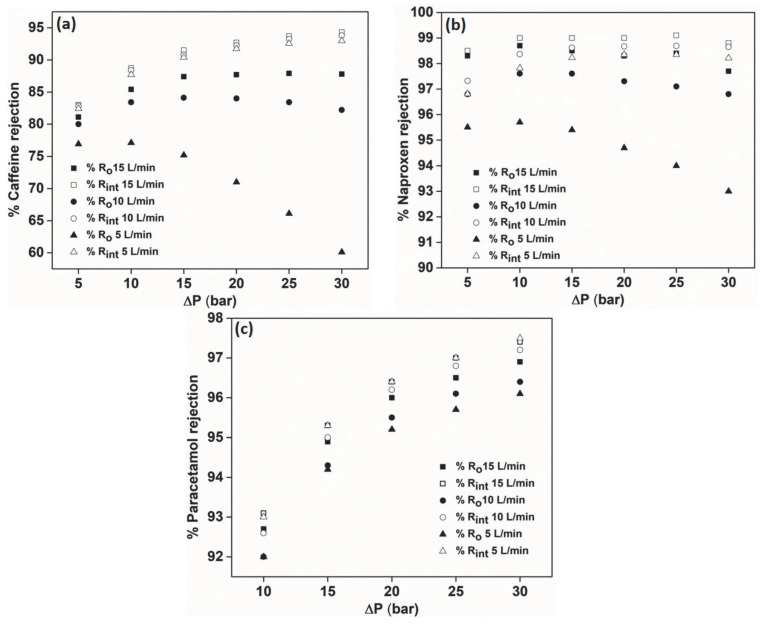
Effect of feed flow rate on the rejection efficiency of (**a**) caffeine, (**b**) naproxen and (**c**) paracetamol.

Figure 7 illustrates the effect of feed flow rate on the rejection efficiency of caffeine and naproxen by the AFC 40 membrane and paracetamol by the AFC 80 membrane. The maintained feed flow rate of 5 to 15 dm^3^/min corresponds to a cross-flow velocity (*u*) of 0.7–2 m s^−1^. In this study, unlike in previous cases, a huge impact was seen on the observed rejection. To get a clear picture of this noteworthy outcome, the intrinsic rejection (which takes account of the concentration polarisation at the membrane surface) was also compared with the observed rejection values. It was noticed that lowering the feed flow rate led to the decrease, as well as reversing the trend of rejection, where rejection for caffeine and naproxen decreased with increasing transmembrane pressure. However, this trend was not seen for the intrinsic rejection values at 10 dm^3^/min and 5 dm^3^/min feed flow rate. Thus, it is clear that the intrinsic rejection is independent of the feed flow rate, and the rejection increases with transmembrane pressure, as observed in all the other cases discussed above. Nevertheless, the trend obtained for the observed rejection can be explained with the help of a phenomenon called the ‘shielding effect’, caused by the solute ions present at the membrane surface on the feed side.

The solute ions tend to form a layer in front of the negatively charged membrane surface, causing concentration polarisation. This effect is enhanced at lower feed flow rates as the velocity of the feed stream is reduced, and the ions get more time to become clogged in the pores of the membranes. Thus, this effect becomes more pronounced at a 5 dm^3^/min feed flow rate and was observed for the AFC 40 membrane. In contrast, in the case of paracetamol rejection, which was carried out using the AFC 80 membrane, the ions cannot get clogged in the membrane pores due to the smaller pore size and are unaffected by the low feed flow rate. This concentration polarisation phenomenon becomes more pronounced as the transmembrane pressure rises, providing the ions with better momentum to become adhered into the pores.

Therefore, it can be affirmed that a smaller pore size membrane is prone to concentration polarisation, affecting its performance. In general, for dense membranes (AFC 80), concentration polarisation can be almost negligible, but, in a loose membrane (AFC 40), it is more prominent since it has a higher flux owing to bigger pores. This is also confirmed by the unimpeded permeate flux (L_p_) of the AFC 80 (Table 4) in comparison to the slight decrease of the permeate flux with decreasing feed flow rate for caffeine and naproxen rejection by the AFC 40 membrane. This result suggests that dense NF membranes are more suitable for drug separation due to lower fouling tendency and can be applied for different drugs which are not easily separated by loose NF membranes.

### 3.7. Mathematical Modelling

**Figure 8:**
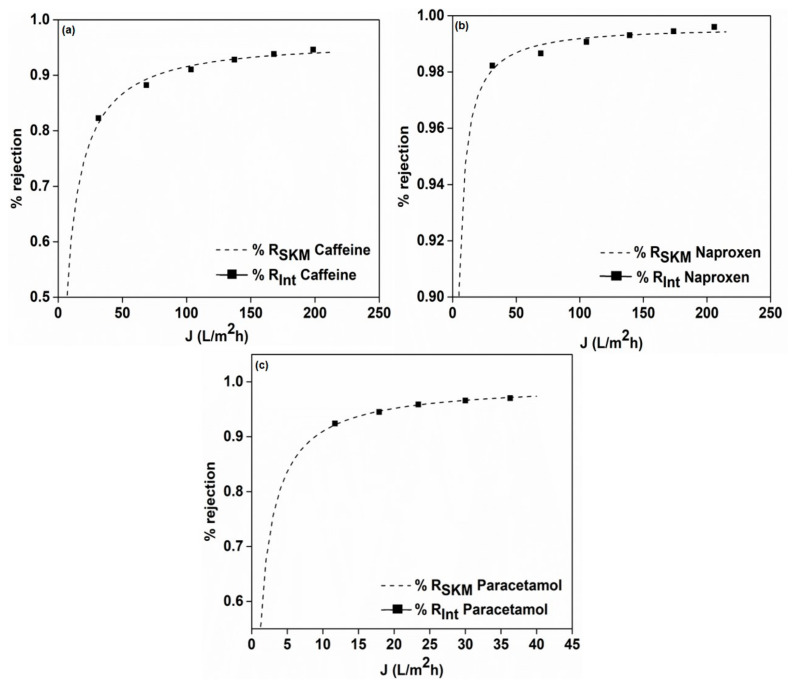
Spiegler–Kedem model fit for the intrinsic rejection values of (**a**) paracetamol, (**b**) caffeine and (**c**) naproxen.

Figure 8 depicts the Spiegler–Kedem model fit for the rejection of caffeine, naproxen and paracetamol, where a perfect fit was observed for the intrinsic rejection (R_int_) and model rejection (R_SKM_) values. The Spiegler–Kedem model is based on the principle of reverse thermodynamics, where the membrane is considered as a black box.

Table 5 illustrates the model fit parameters with a high reflection coefficient in all three cases (>0.95), signifying that the chosen membrane had high retention for the separated drug. The low χ^2^ (which denotes the difference between the experimental and model data values) implies the accuracy of the model in predicting separation patterns. Furthermore, all the data are in coherence with results obtained from our previous studied drug residues [29]. Hence, it proved its usability as a tool to predict any separation outcome, which can be helpful for the application of such NF membranes in real wastewater treatment scenarios. This study, thus, opens up new directions, providing the basis for selecting specific membranes for certain drugs depending on their chemical characteristics and the possibility of predicting fouling and rejection patterns. 

## 4. Conclusions

In summary, it can be concluded that both NF membranes (namely the AFC 40 and AFC 80) were successfully employed in the separation of different drug residues based on pore size and retention efficiency for drugs, dependent on their molecular weight. The separation mechanism predominantly followed the sieving effect; hence, the AFC 40 membrane was ineffective in retaining paracetamol (MW = 151.16 g/mol) due to its larger pore size of 0.345 nm. However, the AFC 40 was able to retain caffeine and naproxen with rejection rates of 88% and 98%, respectively. In optimum conditions, the AFC 80 membrane with a smaller pore size of 0.283 nm rejected 96% paracetamol.

The results are competitive with several other studies that reported the use of different NF membranes to remove such drugs. For example, Mahlangu et al. [33] were able to remove caffeine with 85% rejection using NF 270 membrane. Similarly, Licona et al. [34] and Hidalgo et al. [35] separated caffeine using BW 30 and NF 99 membranes resulting in ~95% and 84% rejection, respectively. The NF 270 membrane also showed good rejection of paracetamol of about 84%, as described by Garcia-Ivars et al. [18]. However, the same NF 270 membrane was also reported to give a rejection of 31%, and the NF 90 membrane gave 91% [36]. With such diverse data available, mainly focusing on NF 270, NF 90 and NF 99 membranes, the AFC 40 and 80 membranes are considered a good alternative for the removal of pharmaceutical drug residues and can also be applied for the removal of other PhACs with similar physio-chemical properties to the drugs used in this study.

The present study demonstrates the effectiveness of the commercial NF membrane AFC 40 in the removal of caffeine and naproxen and the AFC 80 for the removal of paracetamol. In the optimum conditions of maximum feed flow rate (15 dm^3^/min), external operating transmembrane pressure causes an overall increase in flux, as well as rejection. The AFC 40 membrane belongs to the loose NF category, with suitability for the removal of caffeine and naproxen due to its larger molecular weight. The AFC 80 membrane belongs to the dense NF category, with suitability for the removal of paracetamol.

The overall separation mechanism is dominated by the sieving effect. The effect of a lower feed flow rate causes maximum concentration polarisation, which sacrifices the rejection efficacy, whereas the presence of NaCl solute decreases the permeate flux.

Furthermore, the AFC 80 membrane is least affected by the external operating parameters, such as flow rate, feed concentration and ionic strength, due to its inherent, smaller pore size. Therefore, the Spiegler–Kedem model applied in studying the rejection pattern of the membranes provides a valuable insight in the rejection phenomenon at the membrane surface and can be a beneficial tool for evaluating future possibilities for practical separation experiments.

## Figures and Tables

**Table 1:** Characteristics of the NF membranes used.

Structural Features	AFC 40	AFC 80
Membrane type	Tubular	Tubular
Material/Substrate	Polyamide selective layer/polysulfone	Polyamide selective layer/polysulfone
Usable pH range	1.5–10.5	1.5–10.5
Maximum pressure (bar)	60	60
Maximum temperature (°C)	60	70
Surface charge	Negative	Negative
Effective membrane area (cm^2^)	240	240
Membrane length (cm)	30	30
Internal diameter (cm)	1.25	1.25

**Table 2:** HPLC parameters for caffeine, paracetamol and naproxen.

HPLC Parameters	Mobile Phase (Water/Acetonitrile) (%)	Sample Injection Volume (µL)	Flow Rate (mL/min)	Detector (nm)	Retention Time (R_t_) (min)
Caffeine	55/45	10	1	λ_max_: 274	2.35
Paracetamol	55/45	10	1	λ_max_: 248	2.22
Naproxen	30/70	10	0.6	λ_max_: 232	4.82

**Table 3:** Classification and physio-chemical properties of the selected pharmaceuticals.

Drug	MW (g/mol)	Chemical Formula	Solubility	Classification	Charge	Structure
Caffeine	194.9	C_8_H_10_N_4_O_2_	Water	Central nervous system stimulant	Neutral	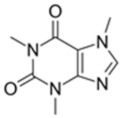
Naproxen	230.2	C_14_H_14_O_3_	Water and organic	Anti-inflammatory, analgesic	Negative	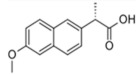
Paracetamol	151.16	C_8_H_9_NO_2_	Water	Analgesic, antipyretic	Partially negative or neutral	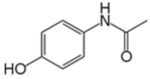

**Table 4:** Hydraulic coefficients of the AFC 40 and AFC 80 membranes obtained at different feed flow rates during PhAC separation.

Experiment	Hydraulic Permeability Coefficient (L_p_)		
Feed Flow Rate (L/min)	Caffeine	Naproxen	Paracetamol
5	6.33	5.38	1.22
10	6.64	5.95	1.25
15	6.80	6.21	1.24

**Table 5:** Model parameters (σ, ω) and errors (χ^2^) of Spiegler–Kedem model for experiments with caffeine, naproxen and paracetamol.

Experiment		AFC 40			AFC 80		
Solute	Conc. (mg/L)	Σ (-)	ω (Lm^−2^h^−1^)	χ2 (-)	Σ (-)	ω (Lm^−2^h^−1^)	χ2 (-)
Caffeine	20	0.952	1.67 × 10^−6^	3.19 × 10^−4^	Not carried out since high rejection obtained with the AFC 40 membrane		
Naproxen	20	0.995	1.46 × 10^−7^	1.74 × 10^−5^	Not carried out since high rejection obtained with the AFC 40 membrane		
Paracetamol	20	Very low rejection obtained			0.995	2.66 × 10^−7^	1.07 × 10^−5^

## Data Availability

Not applicable.
